# Angiotensin II type 1 receptor blockers increase tolerance of cells to copper and cisplatin

**DOI:** 10.15698/mic2014.11.175

**Published:** 2014-10-24

**Authors:** Pieter Spincemaille, Gursimran Chandhok, Andree Zibert, Hartmut Schmidt, Jef Verbeek, Patrick Chaltin, Bruno P. Cammue, David Cassiman, Karin Thevissen

**Affiliations:** 1 Centre of Microbial and Plant Genetics (CMPG), KU Leuven, Kasteelpark Arenberg 20, 3001 Heverlee, Belgium.; 2 Clinic for Transplantation Medicine, Münster University Hospital, Albert-Schweitzer-Campus 1, Building A14, D-48149 Münster, Germany.; 3 Department of Hepatology and Metabolic Center, University Hospital Gasthuisberg, Herestraat 49, 3000 Leuven, Belgium.; 4 CISTIM Leuven vzw, Bio-Incubator 2, Wetenschapspark Arenberg, Gaston Geenslaan 2, 3001 Heverlee, Belgium.; 5 Centre for Drug Design and Discovery (CD3), KU Leuven R&D, Waaistraat 6, Box 5105, 3000 Leuven.; 6 Department of Plant Systems Biology, VIB, Technologiepark 927, 9052, Ghent, Belgium.

**Keywords:** sartans, copper, cisplatin, drug repositioning

## Abstract

The human pathology Wilson disease (WD) is characterized by toxic copper (Cu) accumulation in brain and liver, resulting in, among other indications, mitochondrial dysfunction and apoptosis of hepatocytes. In an effort to identify novel compounds that can alleviate Cu-induced toxicity, we screened the Pharmakon 1600 repositioning library using a Cu-toxicity yeast screen. We identified 2 members of the drug class of Angiotensin II Type 1 receptor blockers (ARBs) that could increase yeast tolerance to Cu, namely Candesartan and Losartan. Subsequently, we show that specific ARBs can increase yeast tolerance to Cu and/or the chemotherapeutic agent cisplatin (Cp). The latter also induces mitochondrial dysfunction and apoptosis in mammalian cells. We further demonstrate that specific ARBs can prevent the prevalence of Cu-induced apoptotic markers in yeast, with Candesartan Cilexetil being the ARB which demonstrated most pronounced reduction of apoptosis-related markers. Next, we tested the sensitivity of a selection of yeast knockout mutants affected in detoxification of reactive oxygen species (ROS) and Cu for Candesartan Cilexetil rescue in presence of Cu. These data indicate that Candesartan Cilexetil increases yeast tolerance to Cu irrespectively of major ROS-detoxifying proteins. Finally, we show that specific ARBs can increase mammalian cell tolerance to Cu, as well as decrease the prevalence of Cu-induced apoptotic markers. All the above point to the potential of ARBs in preventing Cu-induced toxicity in yeast and mammalian cells.

## INTRODUCTION

The human pathology Wilson disease (WD) is characterized by excess copper (Cu) accumulation in the brain and liver, leading to liver failure or cirrhosis and neurodegeneration 1,2,3. Cu toxicity is directly related to mitochondrial dysfunction and apoptosis in mammalian cells 4 as it induces oxidative stress 5,6 and crosslinking of mitochondrial membrane proteins causing the membrane to contract 7. In addition, Cu causes a malfunction of complex IV of the respiratory chain 8 and increases acid sphingomyelinase (aSMase) activity 9, the latter leading to an increased production of the apoptosis inducer ceramide 10. Furthermore, the chemotherapeutic agent cisplatin (Cp) also induces mitochondrial dysfunction in mammalian cells by decreasing respiration 11, causing mitochondrial membrane depolarization 12, inducing the production of reactive oxygen species (ROS) 13, and affecting mitochondrial structure and function 14. In addition, mitochondria generate ROS upon exposure to Cp in both yeast and mammalians cells 13, indicating that Cp-induced toxicity shows similarity between yeast and mammalian cells. In yeast, there are several contradictory reports on Cu-induced toxicity and mitochondrial dysfunction. Some reports have demonstrated the negative impact of non-lethal Cu doses on the mitochondrial proteome and function in yeast, resulting in decreased ATP production and activation of the oxidative stress response 15, and mitochondrial abnormalities 16. In contrast, Cu-treatment of yeast has also been reported to increase the mitochondrial Cu content without causing respiratory deficits 17. Apart from direct effects of Cu on mitochondrial function, Cu toxicity in *S. cerevisiae* has been linked to perturbations in sphingolipid (SL) homeostasis 18, which are crucial membrane components with regard to apoptosis 19 and mitochondrial function 20,21. In contrast, Lee and coworkers did not show any alterations in a subset of SL species in response to Cu treatment 22. Hence, the question whether Cu indeed results in mitochondrial dysfunction in yeast remains under debate. Nonetheless, by using a Cu-induced toxicity screen in yeast, we previously identified an *Arabidopsis thaliana*-derived decapeptide termed OSIP108 23 as a peptide that can increase yeast tolerance to Cu. More specifically, we showed that OSIP108 prevents Cu-induced apoptosis in yeast and human cells, and preserves mitochondrial ultrastructure in human cells 18. Furthermore, we were able to link these observations to perturbations in SL homeostasis by OSIP108 18. In addition, we translated these data toward a novel zebrafish model for Cu toxicity and showed that OSIP108 injections into zebrafish larvae prevented Cu-induced hepatotoxicity and decreased oxidative stress levels 24. Thus, despite the contradictory reports in literature, by using our Cu-toxicity yeast screen, we identified OSIP108, and were able to translate our yeast data to higher eukaryotic cell models, as well as to an *in vivo* model for Cu-intoxication, thereby validating our Cu-toxicity screen in yeast in the context of apoptosis and mitochondrial dysfunction.

In an effort to identify small molecules that can alleviate Cu-induced toxicity in yeast, we screened the Pharmakon 1600 repositioning library consisting of 1600 drugs, which are marketed or have been tested in clinical trials. Drug repositioning is referred to as the identification and development of new uses of existing or abandoned drugs. It possesses several advantages over *de novo* drug discovery such as known safety and pharmacokinetic profiles, as well as knowledge of manufacturing and toxicology of the compounds investigated 25,26. Current fields of interest for application of such repurposing strategy include the identification of novel antibiotics 27, the increase of effectiveness of existing antimycotics by potentiation 28, but also novel treatments for orphan diseases 29. The Pharmakon library was screened in our Cu-based yeast-toxicity screen 18. Repurposed compounds that scored positive in this Cu-based yeast toxicity screen were further tested for their potential to increase yeast tolerance to Cp, another inducer of mitochondrial dysfunction. Subsequently, we translated these data to a mammalian cell setting. All our data point to the protective effect of ARBs against Cu-induced toxicity.

## RESULTS 

### Screening for compounds that can increase yeast tolerance to Cu

The Pharmakon 1600 repositioning library was screened for agents that can increase yeast tolerance to Cu as described previously 18. Briefly, WT yeast was inoculated in solid growth medium containing a lethal Cu concentration (100 µM) and the viability indicator dye MTT (0.1 mg/mL). All 1600 compounds (10 mM in DMSO) were spotted (5 µL) onto the solid agar. Following 24 h of incubation, the plates were checked for development of purple halos around the spotted compounds, resulting from the conversion of the viability dye MTT and thus indicative for viable cells. Given that Cu chelation or sequestration is one of the main cellular Cu detoxification mechanisms 30,31, we identified several agents with known chelating activity such as Deferoxamine Mesylate 32 and Oxyquinoline Sulfate 33. Hence, such agents were omitted to exclude aspecific Cu chelation. This resulted in the identification of seven compounds (data not shown) that are not known to chelate Cu and can increase tolerance of yeast cells to Cu. Among them were two members of the drug class of Angiotensin II Type 1 receptor blockers (ARBs) 34, namely Candesartan and Losartan.

Given the fact that several studies have documented beneficial effects of ARBs, such as Candesartan and Losartan, on human pathologies linked to mitochondrial dysfunction and apoptosis, such as diabetes, Alzheimer disease and aging 35,36,37,38,39,40,41,42, we selected the drug class of ARBs for further characterization of their activity using the model yeast *S. cerevisiae*.

### Angiotensin II Type 1 receptor blockers increase yeast survival in presence of toxic Cu and Cp

**Table 1:** Effect of the selected ARBs on Cu and Cp-induced toxicity in yeast. ‘+’ and ‘-‘ denotes effective and ineffective, respectively.

	**Toxicity**	
**ARB**	**Cu**	**Cp**
Candesartan	-	+
Candesartan Cilexetil	+	+
Eprosartan	-	-
Irbesartan	-	+
Losartan	+	+
Olmesartan	-	-
Olmesartan Medoxomil	+	+
Telmisartan	-	-
Trityl Candesartan Cilexetil	-	-
Valsartan	+	-

**Figure 1:**
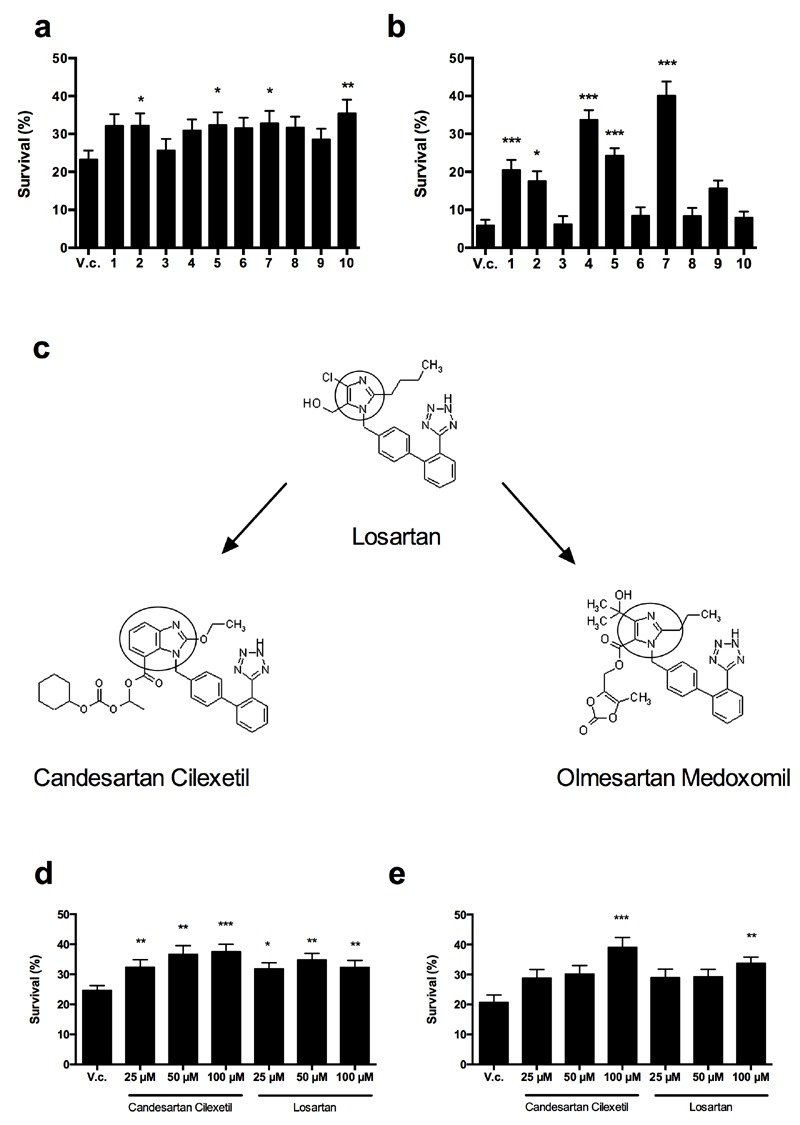
FIGURE 1: ARBs prevent Cu and Cp-induced killing of yeast cells. Yeast cells were incubated with 2 mM Cu for 4 h **(a, d)** or 250 µM Cp for 16 h **(b, e)** in presence of vehicle control (V.c, 2.5% DMSO) or 100 µM Candesartan (1), Candesartan Cilexetil (2), Eprosartan (3), Irbesartan (4), Losartan (5), Olmesartan (6), Olmesartan Medoxomil (7), Telmisartan (8), Trityl Candesartan Cilexetil (9) or Valsartan (10). **(c)** Chemical structure of the prototype Losartan, of which Candesartan Cilexetil and Olmesartan Medoxomil are derived**. **The benzimidazole group in Candesartan Cilexetil and the imidazole ring in Losartan and Olmesartan Medoxomil are circled. Taken and adapted from 44. Yeast survival in presence of different doses of Candesartan Cilexetil or Losartan upon treatment with 2 mM Cu **(d)** or 250 µM Cp **(e)**. Survival was calculated by determining CFU/ml as compared to untreated control cells (no Cu or Cp). Experiment performed in quadruplicate, with at least two biological repeats. (*P < 0.05; **P < 0.01; ***P < 0.001; ANOVA test using Tukey correction).

We investigated the effect of a selection of commercially available ARBs, namely Eprosartan, Irbesartan, Losartan, Olmesartan, Olmesartan Medoxomil, Telmisartan, Trityl Candesartan Cilexetil and Valsartan (Table 1), on yeast survival in presence of toxic Cu and Cp. The prototype of the class of ARBs is Losartan 43, from which additional ARBs were derived based on modification of its chemical structure, such as Candesartan, Valsartan and Olmesartan 34. We found that incubation of yeast cells with 2 mM Cu resulted in approx. 23% yeast survival, whereas treatment of Cu-stressed yeast with either 100 µM Candesartan Cilexetil, Losartan, Olmesartan Medoxomil or Valsartan significantly increased yeast survival. No significant increase in survival of Cu-stressed yeast cells was observed upon incubation with 100 µM Candesartan, Eprosartan, Irbesartan, Olmesartan, Telmisartan or Trityl Candesartan (Fig. 1 a). These results indicate that particular ARBs can increase yeast tolerance to excess Cu. Note that while Candesartan was identified during our screen, it failed to significantly increase yeast survival in presence of Cu, as determined by plating colony-forming units (CFU). It is expected that the CFU-based assay is more stringent, resulting in the selection of the most potent ARBs with regard to increasing Cu tolerance.

We further evaluated the effect of the ARBs against Cp-induced toxicity in yeast. Incubation of yeast cells with 250 µM Cp decreased yeast survival to about 5% whereas co-treatment with either 100 µM Candesartan, Candesartan Cilexetil, Irbesartan, Losartan or Olmesartan Medoxomil significantly increased survival as compared to control treatment. No significant effect on survival of Cp-stressed yeast cells was observed with treatment of 100 µM Eprosartan, Olmesartan, Telmisartan, Trityl Candesartan Cilexetil or Valsartan (Fig. 1 b). These data indicate that particular ARBs can prevent Cp-induced toxicity in yeast.

Interestingly, in our experiments only Candesartan Cilexetil, Losartan and Olmesartan Medoxomil increased yeast tolerance to both Cu and Cp (Table 1), suggesting that these three ARBs are the most interesting candidates. Structural classification of ARBs is based on the cycle replacing the imidazole ring in Losartan: in Candesartan Cilexetil, this imidazole group is replaced by a benzimidazole group, while in Olmesartan Cilexetil it is a imidazole derivate 34. Since these three ARBs significantly increased yeast tolerance to both Cu and Cp (Fig. 1 a, b) and Candesartan Cilexetil is the most structurally different from Losartan (Fig. 1 c), we selected the prototype ARB Losartan and Candesartan Cilexetil for further characterization. For a more detailed description on the chemical structure of ARBs the reader is referred to 34,44.

In order to gain insight into the efficacy of Candesartan Cilexetil and Losartan against Cu or Cp-induced toxicity in *S. cerevisiae*, we evaluated different doses (25 µM - 100 µM) of either ARB on yeast survival in presence of toxic Cu (2 mM) or Cp (250 µM). We found that all tested doses of Candesartan Cilexetil and Losartan significantly increased yeast tolerance to Cu (Fig. 1 d). Conversely, we observed that only 100 µM Candesartan Cilexetil and 100 µM Losartan significantly increased yeast survival in presence of Cp (Fig. 1 e). These data indicate that ARBs can protect yeast against Cu-induced toxicity in a broad concentration range, while only high doses of ARBs confer protection against Cp-induced toxicity.

In order to exclude a general stress-protectant effect of ARBs, we also evaluated the effect of ARBs against a panel of noxious insults such as tunicamycin, the anti-convulsant valproic acid, acetic acid and CCCP. To this end we monitored yeast growth in presence of either insult in absence (control) or presence of 100 µM Candesartan Cilexetil and/or Losartan. In contrast to the effect of ARBs on yeast growth in presence of Cp, we did not observe an effect of the tested ARBs against either insult (Supplemental Fig 1 a-e). These results suggest that ARBs cannot rescue general yeast growth defects induced by toxic triggers including tunicamycin, valproic acid, acetic acid or CCCP and hence, that the ARB-protecting action against noxious insults seems toxin-specific.

### ARBs affect Cu-induced apoptotic markers in yeast 

**Figure 2:**
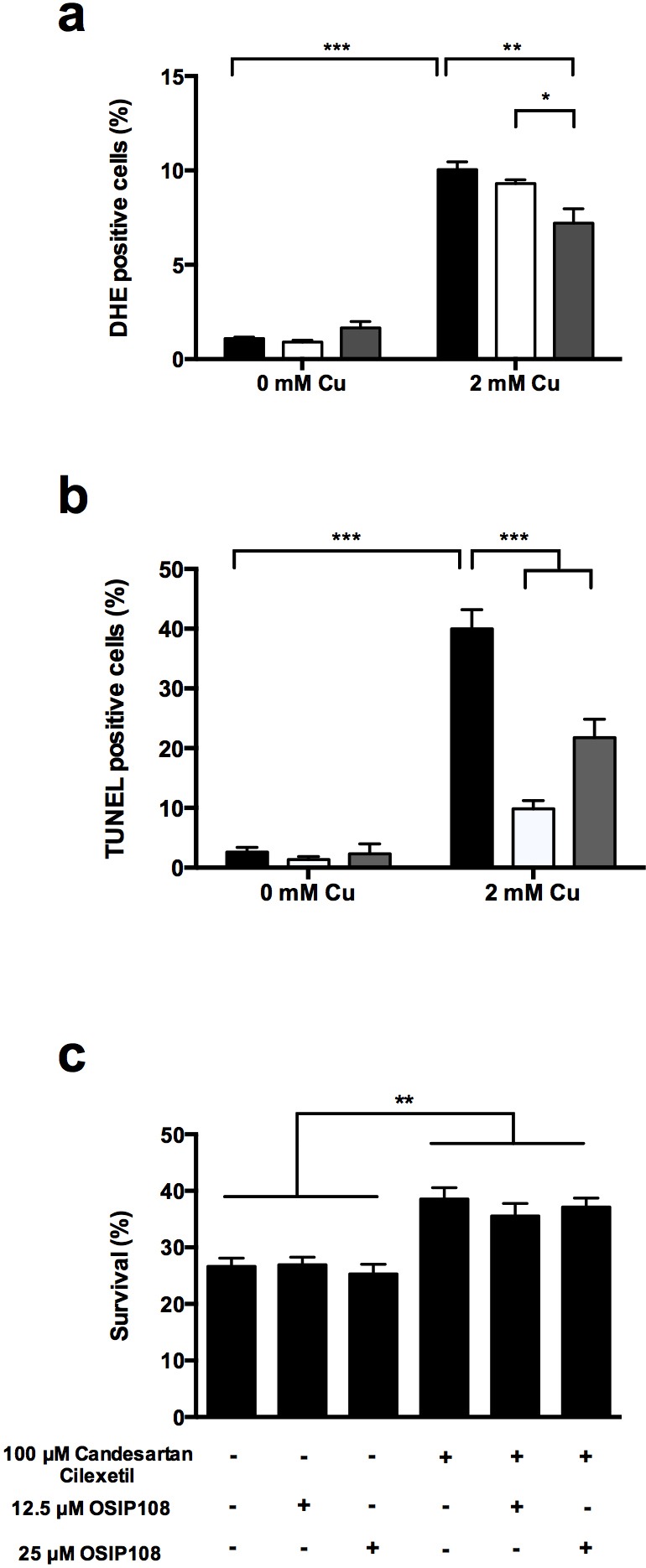
FIGURE 2: ARBs reduce levels of Cu induced apoptotic markers in yeast. Yeast cells were incubated with 2 mM Cu for 4 h with control treatment (black bars), Losartan (white bars) or Candesartan Cilexetil (gray bars). Following treatment, cells were stained with DHE **(a)** or TUNEL assay **(b)** and subsequently analyzed by flow cytometry or counted manually after fluorescence microscopy, respectively. Three biological repeats were used. (*P < 0.05; **P < 0.01; ***P < 0.001; ANOVA test using Tukey correction). **(c)** OSIP108 does not affect the rescue effect of Candesartan Cilexetil. Yeast cells were incubated with 2 mM Cu for 4 h with control treatment, Candesartan Cilexetil (100 µM) in absence (2% DMSO) or presence of OSIP108 (12.5 µM - 25 µM). Following incubation, cells were plated on YPD agar plates and survival was calculated as compared to CFU/ml of cells receiving no Cu. Experiments performed in quadruplicate with three biological repeats. (*P < 0.05; **P < 0.01; ***P < 0.001; ANOVA test using Tukey correction).

As excess Cu is known to induce apoptosis 18,45, we investigated the effect of Losartan and Candesartan Cilexetil on Cu-induced ROS production and DNA fragmentation, both markers of apoptosis 46. In line with our previous results 18, we observed that 2 mM Cu significantly increased the amount of cells stained positive with dihydroethidium (DHE) (Fig. 2 a), indicating the induction of ROS and more specifically superoxide by Cu. Co-incubation with 100 µM Candesartan Cilexetil significantly decreased the levels of DHE positive cells in presence of 2 mM Cu (Fig. 2 a). By using the Terminal dUTP Nick End Labeling (TUNEL) assay, we observed that 2 mM Cu significantly induced DNA fragmentation in yeast, while treatment with either 100 µM Candesartan Cilexetil or Losartan significantly decreased the amount of Cu-induced TUNEL positive cells (Fig 2 b). These data suggest that specific ARBs may differentially affect Cu-induced markers of apoptosis, and that Candesartan Cilexetil is the most potent in decreasing these markers.

In order to increase our understanding of Cu-induced cell death in yeast, which occurs presumably via apoptosis 18,45, we evaluated the Cu-tolerance and the rescuing effect of ARBs of wild type yeast (BY4741) and a selection of yeast mutants defective in components of the apoptotic machinery such as Δ*aif1*, Δ*nuc1 *or Δ*yca1 *mutants. To this end, we spotted serial dilutions of these yeast cultures onto Cu-containing (1.25 mM - 1.5 mM) solid growth media in the presence of control (0.5% DMSO) or Candesartan Cilexetil (100 µM)*.* This selection of mutants was also recently tested to characterize cell death induced by *Mentha piperita *essential oil in *S. cerevisiae* 47. Aif1p and Nuc1p are mitochondrial cell death effectors that translocate to the nucleus in response to apoptotic stimuli such as hydrogen peroxide 48,49. Yca1p is the yeast metacaspase and is implicated as a crucial cell death effector during H_2_O_2_- or acetic acid-induced apoptosis 50,51. We observed that the *Δyca1* mutant did not display altered susceptibility to toxic Cu as compared to wild type yeast, which is in line with literature 18,45,52, nor did *Δaif1* or* Δnuc1 *mutants (Supplemental Fig. 2, middle and right panel). Candesartan Cilexetil supplementation, however, increased viability of all yeast mutants (Supplemental Fig. 2, middle and right panels). Taken together, these data suggest that Cu-induced apoptosis in yeast is independent of Aif1p, Nuc1p and Yca1p, and that Candesartan Cilexetil increases yeast tolerance to Cu independently of the latter three proteins.

### Exogenous addition of the plant decapeptide OSIP108 does not affect the protective effect of Candesartan Cilexetil against Cu-induced toxicity in yeast

As we previously identified the *A. thaliana*-derived decapeptide OSIP108 23 as a potent rescuer from Cu toxicity of yeast and human cells 18, we evaluated whether OSIP108 can influence the effect of Candesartan Cilexetil on Cu-induced cell death in yeast. Thus, we investigated a putative synergy between Candesartan Cilexetil and OSIP108.

To this end, we first identified an OSIP108 dose that did not affect yeast Cu-tolerance, being 12.5 µM or 25 µM OSIP108 (Fig. 2 c). Subsequently, we found that these OSIP108 doses neither increased nor decreased the protective effect of Candesartan Cilexetil (100 µM) against Cu-induced toxicity in yeast (Fig. 2 c), suggesting that the structurally unrelated OSIP108 and Candesartan Cilexetil affect yeast tolerance to Cu via distinct pathways.

### Candesartan Cilexetil increases yeast mutant tolerance to Cu

To get preliminary insights into the role of ROS during Cu-induced toxicity in yeast, we evaluated the Cu tolerance of a panel of yeast deletion mutants defective in ROS detoxification (Δ*cta1*, Δ*ctt1*, Δ*grx5, *Δ*sod1*, Δ*sod2*), at least partly defective in cytoplasmic ROS production (Δ*yno1*) or Cu sequestration (Δ*crs5*, Δ*cup2*). The catalases Cta1p and Ctt1p detoxify the ROS H_2_O_2 _in the peroxisomes and cytosol, respectively 53,54. During respiratory growth conditions Cta1p also resides in the mitochondria 54. In addition, the superoxide dismutases Sod1p and Sod2p detoxify superoxide, thereby generating oxygen and H_2_O_2_, mainly in the cytosol and mitochondria, respectively 55,56. Furthermore, we included yeast mutants defective in Grx5p, a mitochondrial glutathione-dependent oxidoreductase that plays a crucial role in defense against oxidative stress 57, and Yno1p, a NADPH oxidase in the endoplasmic reticulum that is responsible for extra-mitochondrial superoxide production 58. In addition, given that Cu sequestration is one of the major Cu-detoxification mechanisms, we included the Cu-binding metallothionein Crs5p 59 and the Cu-binding transcription factor Cup2p, which initiates transcription of the genes encoding the metallothioneins *CUP1-1 *and *CUP1-2* 60,61. Serial dilutions of wild type (BY4741) and these mutants were spotted onto control (0 mM Cu) or Cu-containing (1.25 mM - 1.5 mM) solid growth media supplemented with either control (0.5% DMSO) or Candesartan Cilexetil (100 µM).

**Figure 3:**
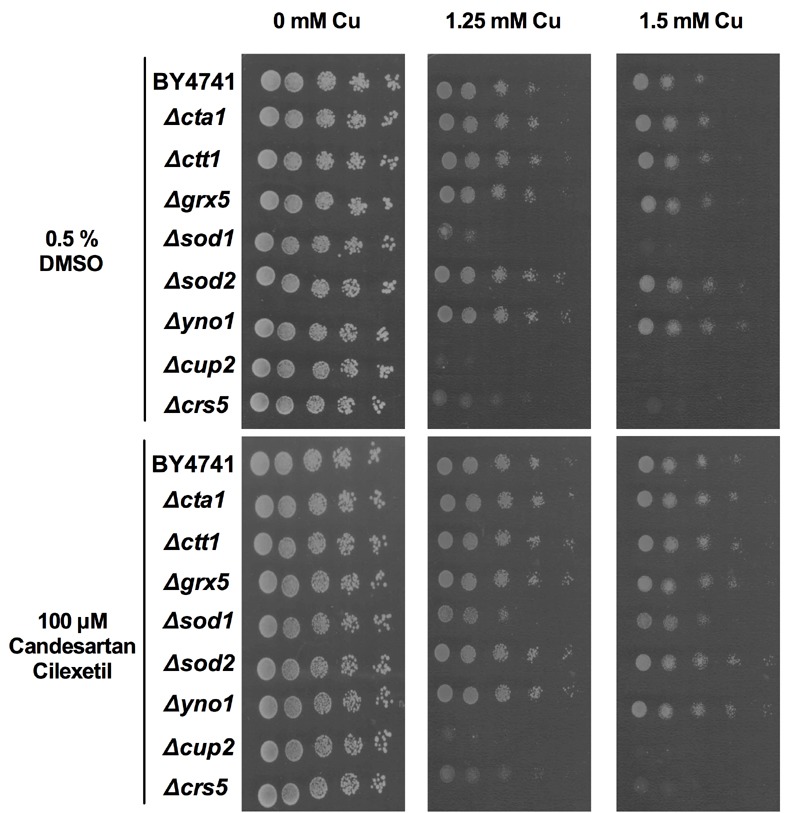
FIGURE 3: Effect of Candesartan Cilexetil on Cu tolerance of yeast mutants. Serial dilutions of wild type (WT), Δ*cta1*, Δ*ctt1, *Δ*grx5, *Δ*sod1, *Δ*sod2, *Δ*yno1, *Δ*cup2 *and Δ*crs5 *were spotted onto control (0 mM Cu) or Cu-containing (1.25 mM - 1.5 mM) solid SC media in presence of control (0.5% DMSO; top panels) or 100 µM Candesartan Cilexetil (bottom panels). Growth was evaluated following 48 h incubation at 30°C. Data representative for two biological repeats.

We found that while loss of Sod1p, Cup2p or Crs5p severely increased yeast sensitivity to Cu, loss of Sod2p or Yno1p moderately increased yeast tolerance to Cu (Fig. 3, middle and right panels). Loss of Sod2p was already previously shown to increase yeast tolerance to copper nitrate 62 while loss of Yno1p is documented to increase yeast tolerance to apoptotic stimuli 58. In contrast, supplementation with Candesartan Cilexetil increased the Cu tolerance of all yeast mutants, except Δ*crs5* and Δ*cup2 *mutants. Given the evident increased susceptibility to Cu of the latter two mutants, this suggests that both proteins are crucial in conferring Cu tolerance in yeast and their loss is detrimental for yeast viability in presence of Cu to an extent that Candesartan Cilexetil is unable to counteract this toxicity. However, additional experiments are required to determine whether Candesartan Cilexetil directly acts on metallothionein levels/activity. Surprisingly, given the increased and decreased susceptibility of Δ*sod1 *and Δ*yno1* mutants to Cu, respectively, this points to deleterious cytoplasmic, rather than mitochondrial, ROS production during Cu-induced toxicity in yeast. However, this does not exclude mitochondrial ROS production upon Cu treatment in yeast. Note that Cu is an essential cofactor for Sod1p, and loss of this protein may trigger defective Cu storage. Taken together, further research is needed to pinpoint the cellular targets of Candesartan Cilexetil in the context of Cu toxicity in yeast.

### ARBs prevent Cu-induced apoptosis in the human hepatoma HepG2 cell line 

**Figure 4:**
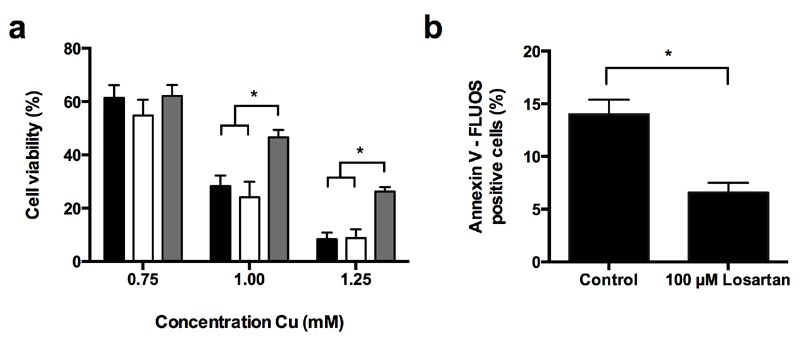
FIGURE 4: Losartan prevents Cu-induced apoptosis in HepG2 cells. **(a)** Losartan increases HepG2 cell viability in presence of Cu. HepG2 cells were treated with Cu (0.75 mM - 1.25 mM) in presence of control (1% DMSO, black bars), 25 µM Candesartan Cilexetil (white bars) or 100 µM Losartan (grey bars). Following 48 h of incubation, cell viability was determined by MTT viability staining, and expressed as compared to cells receiving no Cu. Biological repeat is four. (***P <0.001; ANOVA test using Tukey correction). **(b)** Losartan prevents Cu-induced apoptosis in HepG2 cells. HepG2 cells were treated for 1.5 mM Cu in absence (control) or presence of 100 µM Losartan for 24 h. Subsequently, cells were stained with FLUOS-labeled Annexin V and samples were analyzed by flow cytometry. Biological repeats is three. (**P < 0.01; Student t-test).

In an effort to translate our yeast data to a higher eukaryotic cell model, we investigated the effect of the ARBs Candesartan Cilexetil and Losartan on Cu-induced apoptosis in the human hepatoma HepG2 cell line. To this end we first evaluated HepG2 survival in absence of Cu upon incubation with Candesartan Cilexetil and Losartan. In initial experiments we titrated the Candesartan Cilexetil or Losartan dose to investigate a putative ARB-associated toxicity towards HepG2 cells, and observed that while doses higher than 25 µM Candesartan Cilexetil induced killing of HepG2 cells, 100 µM Losartan did not have an effect on cell viability (data not shown). Subsequently, we investigated the effect of 25 µM Candesartan Cilexetil and 100 µM Losartan on HepG2 cell viability in presence of different Cu concentrations. While ARB treatment in presence of Cu doses that decreased cell viability by less than 50% did not result in any significant effects (Fig. 4 a), we found that only 100 µM Losartan significantly increased cell viability upon treatment with 1 mM or 1.25 mM Cu (Fig. 4 a): HepG2 viability decreased to approx. 28% or 8% upon treatment with 1 mM or 1.25 mM Cu, respectively, while coincubation with 100 µM Losartan resulted in 46% or 26% viability respectively. Coincubation with 25 µM Candesartan Cilexetil and Cu did not affect cell viability. These data suggest that particular ARBs confer protection against severe Cu-induced cell death in mammalian cells.

Subsequently, similar as to our yeast experiments, we evaluated the effect of Candesartan Cilexetil and Losartan on Cu-induced apoptosis in HepG2 cells by FLUOS-labeled Annexin V staining. In line with literature 9,18, we observed an increased prevalence of Annexin V-positive cells upon treatment with 1.5 mM Cu, indicating Cu-induced apoptosis (Fig. 4 b). In addition, while treatment with 100 µM Losartan decreased Cu-induced apoptosis (Fig. 4 b), 25 µM Candesartan Cilexetil did not (data not shown). Taken together, these data indicate that specific ARBs can prevent Cu-induced cell death and apoptosis in human cells.

## DISCUSSION

In the present study we report on the screening of the Pharmakon 1600 repositioning library in order to identify agents that increase tolerance to Cu-induced toxicity in yeast and the identification of the drug class of ARBs. Next, we showed that specific ARBs increase yeast tolerance to Cu and Cp, and affect markers of Cu-induced apoptosis. Likewise, we found that specific ARBs increase human cell line tolerance to Cu and decrease the prevalence of apoptotic markers.

Our Cu-based yeast toxicity screen resulted in the identification of 7 clinically used drugs that can significantly increase yeast tolerance to Cu (data not shown) including the ARBs Candesartan and Losartan that are used as anti-hypertensive drugs 63. The drug class of ARBs is of particular interest given the numerous reports that describe beneficial effects of ARBs in human pathologies related to mitochondrial dysfunction. For instance Losartan treatment reduces mitochondrial dysfunction in aged and spontaneously hypertensive rats 35,36. Furthermore, in a comparative study, Losartan, Olmesartan and Valsartan were shown to prevent liver fibrosis in alloxan-induced diabetic rats. In contrast to Losartan and Valsartan treatment, Olmesartan treatment preserves mitochondrial ultrastructure 39. Even protective effects of ARBs against Alzheimer disease have been suggested 40,41 and long term Losartan administration to rats significantly increases life span 42. Recently, Olmesartan treatment was shown to attenuate high fat diet-induced decreased mitochondrial respiration 64, while Losartan was reported to ameliorate mitochondrial function in indirect flight muscles in *Drosophila melanogaster* 65. An additional advantage of ARBs is that they are well tolerated and not associated with class-related side effects 66,67. In all these studies, however, the underlying molecular mechanism of these beneficial effects of ARBs on mitochondrial function and tolerance to apoptosis were not addressed.

While some ARBs are specific to either Cu or Cp-induced toxicity (Valsartan or Candesartan and Irbesartan, respectively), other ARBs do not show any effect to either toxic insult. However, the ARBs Candesartan Cilexetil, Losartan and Olmesartan Medoxomil display activity against both Cu and Cp (Table 1). Interestingly, Candesartan Cilexetil and Olmesartan Medoxomil are ester prodrug versions of their parent compound Candesartan and Olmesartan, respectively, and display improved bioavailability 68,69. It is therefore plausible that the uptake of these prodrugs in yeast is more efficient, and thereby is a determinant of their protective effect against Cu and Cp. Nonetheless, given the effect of these aforementioned ARBs against both toxic agents, this suggests that Cu and Cp toxicity in yeast may be mediated by overlapping pathways. Common defense mechanisms against Cu and Cp have been described in yeast and mammalian cells. For example, pre-treatment of yeast cells with Cu or Cp is reported to increase yeast tolerance to Cp or Cu, respectively, in both cases due to degradation and delocalization of the Cu transporter Ctr1p 70. Additionally, at low Cu concentrations that do not affect Ctr1p, Cu also protects against Cp-induced toxicity in yeast 71. In mammalian cells, Cu and Cp increase activity of aSMase, leading to an increased production of the apoptosis inducer ceramide 9,72,73 and both Cu and Cp-induced toxicity have also been associated with pro-apoptotic Bax 74,75,76,77,78,79,80. As we observed that ARBs cannot rescue yeast growth defects induced by noxious insults such as tunicamycin, valproic acid, acetic acid or CCCP, it seems that the protective effect of ARBs is toxin-specific.

A Cu-chelating activity has never been reported for ARBs. However, Losartan has been described to spontaneously form an insoluble complex with Cu 81, and ARB-Cu complexes have been reported to display anti-oxidant activity 82,83,84, pointing to the possibility of Cu-chelation by ARBs. Whether the underlying mechanistic event that governs the ARB-mediated protection against Cu-induced toxicity is attributed to Cu complexation by ARBs has yet to be investigated. As Cu-complexation does not explain the ARBs’ effect in Cp toxicity, it is conceivable that ARBs increase yeast tolerance to Cu and Cp in other ways.

With respect to Cp toxicity, there are some *in vivo* reports that describe the effect of ARBs against Cp-induced toxicity. For instance, chronic Losartan treatment in rats after Cp administration improves weight gain following Cp-induced reduced food intake and weight loss 85. Losartan also reduces Cp-induced lipid peroxidation and glutathione depletion in rat kidneys 86. In addition, combination therapies with Losartan and Vitamin E 87 or C 88 as treatment for Cp-induced nephrotoxicity in rats have been tested, but without any significant result. Noteworthy is that both Candesartan and a combination of Candesartan and Cp have been shown to suppress tumor growth in a xenograft model for bladder cancer in mice. However, the combination of Candesartan and Cp proved to be less effective than Candesartan alone, suggesting a protective effect of Candesartan against Cp-induced apoptosis 89.

Yeast is a powerful model organism to study various cellular processes, including mitochondrial function and apoptosis due to conservation of their regulatory pathways 90,91,92,93. Apart from antifungal drug discovery purposes 28,94, yeast-based screens are often used to identify compounds that can affect disease-relevant targets, such as for instance calcineurin 95, human telomerase 96, or synergetic DNA-damaging drug combinations 97. Also, yeast assays have been reportedly used to gain insight into the mode of action of clinically used compounds 98. For instance, the anti-anginal drug Molsidomine 99 was shown to target lanosterol synthase in the sterol biosynthetic pathway 98.

The results of this study indicate that Cu-induced apoptosis in yeast is independent of Aif1p, Nuc1p or Yca1p. In addition, the data presented here also highlight the fact that despite the conservation of several important metabolic pathways, there still remain yeast and mammalian cell-specific aspects: while in mammalian cells Cu-induced toxicity is directly related to impact on mitochondrial function 4,5,6,7,8, there are contradictory reports regarding Cu-induced toxicity and mitochondrial function in yeast 15,16,17. Indeed, the loss of the cytoplasmic ROS generator Yno1p or cytoplasmic Sod1p increases or decreases yeast tolerance to Cu, respectively, indicating that Cu-induced toxicity in yeast seems associated with detrimental cytoplasmic, but does not exclude, mitochondrial ROS production. Still, by using Cu-induced toxicity in yeast as a screening model, we were able to identify the plant-derived peptide OSIP108 as an agent that prevents Cu-induced apoptosis in yeast and human cells, preserves mitochondrial ultrastructure in human cells, but also prevents Cu-induced liver damage and decreases oxidative stress levels in zebrafish larvae 18,23,24. Hence, these reports illustrate the validity of our screen to identify agents with a putative application in persevering mitochondrial function and preventing apoptosis. This is of particular importance as mitochondrial dysfunction, ROS and apoptosis have been linked to several human conditions such as aging, cancer 100, diabetes 101,102 and non-alcoholic steatohepatitis 103, but also neurodegenerative disorders 104 including Alzheimer disease 105 and Parkinson’s disease 106,107, and rare diseases such as WD 4. Current treatment options for mitochondrial dysfunction-related disorders are inadequate and mostly consist of the administration of cofactors and oxygen radical scavengers 108,109,110. Thus, there still is an urgent need for novel treatments in combating mitochondrial dysfunction-related disorders and ARBs may show promise, as well as OSIP108, in this regard.

Noteworthy is that while the effects of ARBs on cellular metabolism in a higher eukaryotic setting is typically based on its ability to block the interaction between Angiotensin II and the Angiotensin II Receptor Type 1, neither the ligand nor the receptor have thus far been identified in *S. cerevisiae, *suggesting the existence of additional cellular targets for ARBs 44,63. Thus, it is plausible that ARBs mediate Cu-tolerance in yeast and mammalian cells irrespective of their known cellular targets. Hence, despite that our yeast data show promise regarding the protective effect of ARBs against Cu-induced toxicity, it therefore remains to be investigated whether their mode of action in yeast can be translated to a mammalian setting.

In conclusion, this study again highlights the potential of *S. cerevisiae* as a model organism, to identify novel compounds that increase tolerance to inducers of mitochondrial dysfunction in mammalian cells such as Cu. Unraveling the anti-Cu mode of action of ARBs in yeast might reveal new therapeutic targets in treatment of WD, or mitochondrial dysfunction-related conditions in general.

## MATERIALS AND METHODS

### Materials and microorganisms

The yeast strains used in this study is *Saccharomyces cerevisiae* wild type yeast strain BY4741 (WT) and corresponding mutants (Δ*aif1, *Δ*crs5, *Δ*cta1, *Δ*ctt1, *Δ*cup2, *Δ*grx5, *Δ*nuc1, *Δ*sod1, *Δ*sod2, *Δ*yca1, *Δ*yno1*) (Euroscarf, Germany) were cultured in SC (0.77 g/L complete amino acid supplement mixture (CSM) (Bio 101 Systems); 6.7 g/L yeast nitrogen base without amino acids (YNB); 20 g/L glucose). HepG2 cells, human hepatoma cells, were purchased from ATCC (Rockville, MD, USA) and grown in Minimal Essential Medium (MEM) supplemented with 10% fetal calf serum, 2 mM L-glutamine, 100 U/ml penicillin and 100 µg/ml streptomycin. Cis-diamminedichloroplatinum (II) (cisplatin, Cp), copper sulphate pentahydrate and copper chloride (CuCl_2_, Cu) were purchased from Sigma-Aldrich (St. Louis, MO, USA) and dissolved in DMSO and distilled H_2_O respectively. Note that there is some controversy in literature regarding the use of DMSO as solvent for Cp. We however did not observe major differences in Cp-induced toxicity in yeast upon using either DMSO or 0.9% NaCl in distilled H_2_O as solvent for Cp. The Pharmakon 1600 repositioning library (10 mM in DMSO) was obtained from MicroSource Discovery Systems, Gaylordsville, CT, USA. All Angiotensin II Type 1 receptor blockers (ARBs) (Candesartan, Candesartan Cilexetil, Eprosartan, Irbesartan, Losartan, Olmesartan, Olmesartan Medoxomil, Telmisartan, Trityl Candesartan Cilexetil and Valsartan, as listed in Table 1) were purchased from Sequoia Research Products (Pangbourne, UK) and dissolved in DMSO. Protocols involving the effect of ARBs on yeast growth in presence of valproic acid, tunicamycin, acetic acid or CCCP are included in the supplementary data.

### Yeast Cu toxicity screen in solid media

The Pharmakon 1600 repositioning library was screened using a Cu-induced toxicity yeast model as described 18.

### Yeast survival in presence of Cu and Cp in liquid media

A WT overnight culture (ONC) in SC was diluted to OD_600_ = 2 in fresh SC and incubated with 2% DMSO (vehicle control) or ARB (25 µM - 100 µM) in presence or absence of 2 mM CuSO_4_ or 250 µM Cp for 4 h or 16 h, respectively. Following incubation at 30°C and 250 rpm, proper cell dilutions were plated onto YPD agar plates (1% Yeast extract, 2% Bacteriological peptone, 2% glucose and 1.5% agar) and survival was calculated as compared to an unstressed yeast culture. Regarding the effect of dhSph or OSIP108 on yeast tolerance to Cu upon treatment with Candesartan Cilexetil, cells were treated as described above in absence (2% DMSO) or presence of OSIP108 (12.5 µM or 25 µM) and 100 µM Candesartan Cilexetil.

### Detection of apoptotic markers in yeast

To determine Cu-induced ROS production Cu-treated yeast cells were stained with 5 µg/ml dihydroethidium (Molecular Probes) (DHE) and analyzed by flow cytometry as described 18. Subsequent data analysis was performed by using FlowJo software (Tree Star Inc., Ashland, MA, USA). For detection of DNA fragmentation, yeast cells were stained by Terminal deoxynucleotidyl transferase dUTP nick end labeling (TUNEL) assay as described 18. TUNEL positive cells were imaged by fluorescence microscopy (Zeiss Axio Imager Z1 fluorescence microscope) and at least 600 cells/samples were manually counted.

### Yeast spot plating

Overnight yeast cultures in SC were diluted to OD_600_ = 0.01 and 5 fold-serial dilutions were spotted on control plates (0 mM CuSO_4_) or Cu-containing (1.25 mM - 1.5 mM) solid SC medium (1.5% agar) in presence of 0.5% DMSO (control) or 100 µM Candesartan Cilexetil. Subsequently, plates were incubated at 30°C for 48 h.

### HepG2 Cu toxicity experiments 

HepG2 viability upon treatment with CuCl_2_ (0.75 mM - 1.25 mM) in presence of 1% DMSO (control), 25 µM Candesartan Cilexetil or 100 µM Losartan was performed as described previously 21. Briefly, 10^4^ cells were seeded in triplicates in 96 well plates and incubated for 24 h. Next day, cells were treated with copper and/or Candesartan Cilexetil or Losartan and further incubated for 48 h. Cell viability was determined by MTT assay and results were calculated as percentage of untreated control cells.

### Detection of apoptosis and oxidative stress in HepG2 cells

Detection of apoptotic markers by FLUOS-Annexin V (Roche Diagnostics NV Belgium, Belgium) staining and subsequent flow cytometry analysis in HepG2 cells upon treatment with 1.5 mM CuCl_2_ in presence of 1% DMSO (control) or 100 µM Losartan was performed as described previously 21. Briefly, 10^6^ cells were seeded in a 6 well plate and incubated for 24 h. Next day, cells were treated with copper and control or Losartan Cilexetil and Losartan for an additional 24 h. Cell culture supernatants and cells were collected and subjected to FLUOS-Annexin V staining followed by flow cytometry analysis (Beckman Coulter, Germany).

## SUPPLEMENTAL MATERIAL

Click here for supplemental data file.

All supplemental data for this article are also available online at http://microbialcell.com/researcharticles/angiotensin-II-type-1-receptor-blockers-increase-tolerance-of-cells-to-copper-and-cisplatin/.
