# Supplementary material for: Angiotensin II type 1 receptor blockers increase tolerance of cells to copper and cisplatin
Source: Microb Cell. 2014 Oct 24;1(11):352–64. doi: 10.15698/mic2014.11.175 (PMC5349125; doi:10.15698/mic2014.11.175)

Supplementary information to:

Angiotensin II Type 1 Receptor Blockers Increase Tolerance of Cells to Copper and Cisplatin

Pieter Spincemaille[1,*], Gursimran Chandhok[2,*], Andree Zibert[2], Hartmut Schmidt[2], Jef Verbeek[3], Patrick Chaltin[4,5], Bruno P.A. Cammue[1,6,†], David Cassiman[3], Karin Thevissen[1,†]

[1]Centre of Microbial and Plant Genetics (CMPG), KU Leuven, Kasteelpark Arenberg 20, 3001 Heverlee, Belgium

[2]Clinic for Transplantation Medicine, Münster University Hospital, Albert-Schweitzer-Campus 1, Building A14, D-48149 Münster, Germany

[3]Department of Hepatology and Metabolic Center, University Hospital Gasthuisberg, Herestraat 49, 3000 Leuven, Belgium.

[4]CISTIM Leuven vzw, Bio-Incubator 2, Wetenschapspark Arenberg, Gaston Geenslaan 2, 3001 Heverlee, Belgium

[5]Centre for Drug Design and Discovery (CD3), KU Leuven R&D, Waaistraat 6, Box 5105, 3000 Leuven,

[6]Department of Plant Systems Biology, VIB, Technologiepark 927, 9052, Ghent, Belgium

[*]Both authors contributed equally to this work

[†]Authors coordinated equally

Corresponding authors:

Bruno P.A. Cammue, Centre for Microbial and Plant Genetics, KU Leuven, Kasteelpark Arenberg 20, 3001 Heverlee, Belgium, Phone: 32-16329682. Fax: 32-16321966. E-mail: bruno.cammue@biw.kuleuven.be

Karin Thevissen, Centre for Microbial and Plant Genetics, KU Leuven, Kasteelpark Arenberg 20, 3001 Heverlee, Belgium, Phone: 32-16329688. Fax: 32-16321966. E-mail: karin.thevissen@biw.kuleuven.be

Running title: Sartans alleviate copper and cisplatin toxicity

Keywords: Sartans, copper, cisplatin, drug repositioning

Supplementary materials and methods

Yeast growth curve determination

An overnight WT yeast culture in SC was diluted to OD600 = 0.01 in fresh SC and incubated with control (2 % DMSO), 250 µM Cp, 2 mM valproic acid, 2.5µM tunicamycin, 8.5 mM acetic acid or 7.81 µM CCCP in absence (2 % DMSO) or presence of 100 µM Candesartan Cilexetil and/or Losartan. Growth was monitored for 36 h by analyzing OD600 with a Bioscreen C MBR (Oy Growth Curves Ab Ltd, Helsinki, Finland). Experiments were performed at 30°C, while shaking continuously at medium amplitude.

Figure legends

Supplementary Fig. 1. ARBs do not trigger a general stress-protectant effect in yeast. Growth of WT yeast was monitored during 36 h upon treatment with 250 µM Cp (a), 2.5 µM tunicamycin (b), 2 mM valproic acid (c), 8.5 mM acetic acid (d) or 7.81 µM CCCP (e) in absence (control, triangles) or presence of 100 µM Candesartan Cilexetil (full circles) and/or 100 µM Losartan (open squares). Growth of an unstressed yeast culture was included as control (open circles). Data is representative for 2 biological repeats.

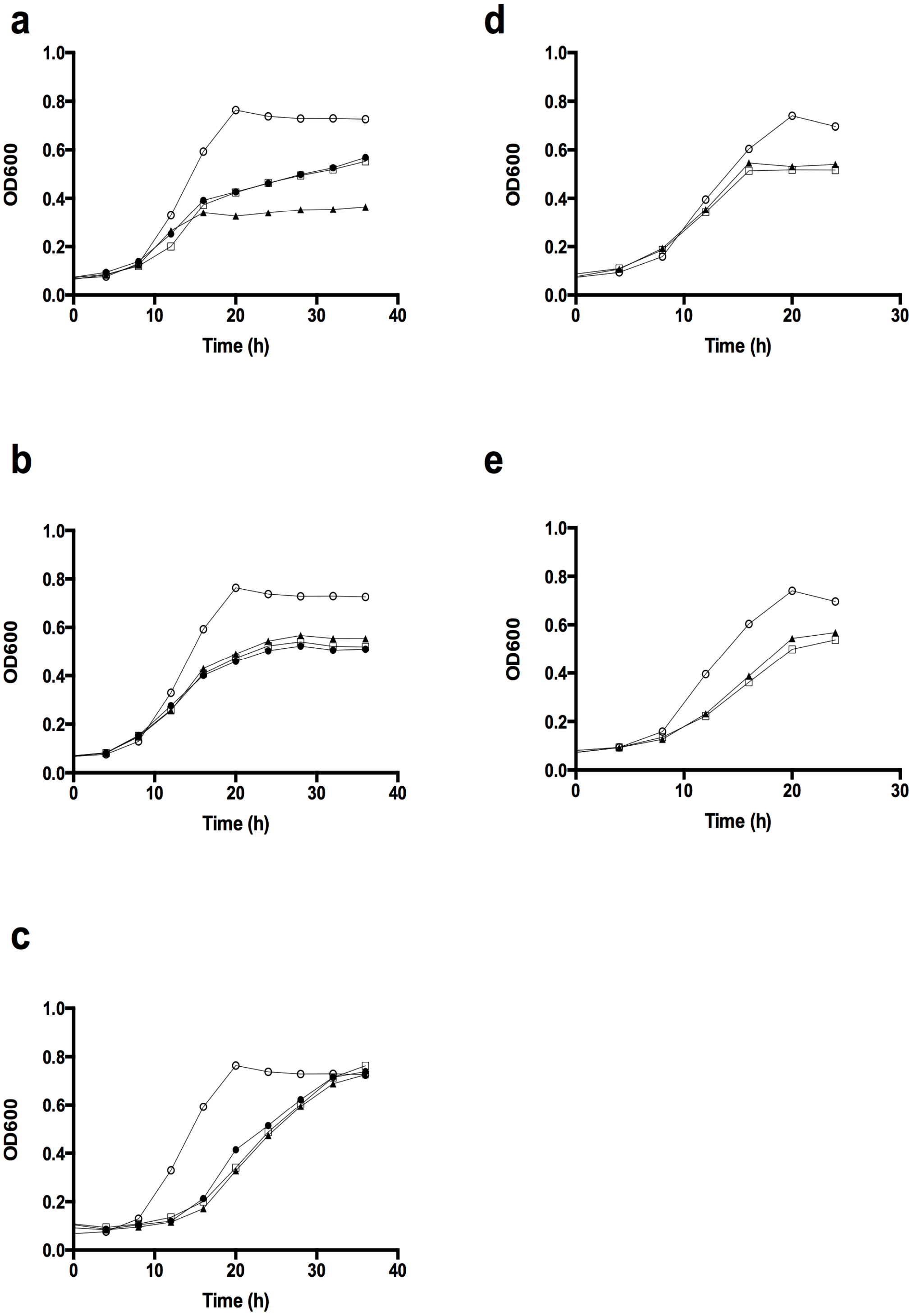

Supplementary Fig. 2. Cu induces Aif1p, Nuc1p and Yca1p-independent cell death. Serial dilutions of wild type (WT), *Δaif1*, *Δnuc1* or *Δyca1* were spotted onto control (0 mM Cu) or Cu-containing (1.25 mM – 1.5 mM) solid SC media in presence of control (0.5 % DMSO; top panels) or 100 µM Candesartan Cilexetil (bottom panels). Cell growth was evaluated following 48 h incubation at 30°C. Data representative for two biological repeats.

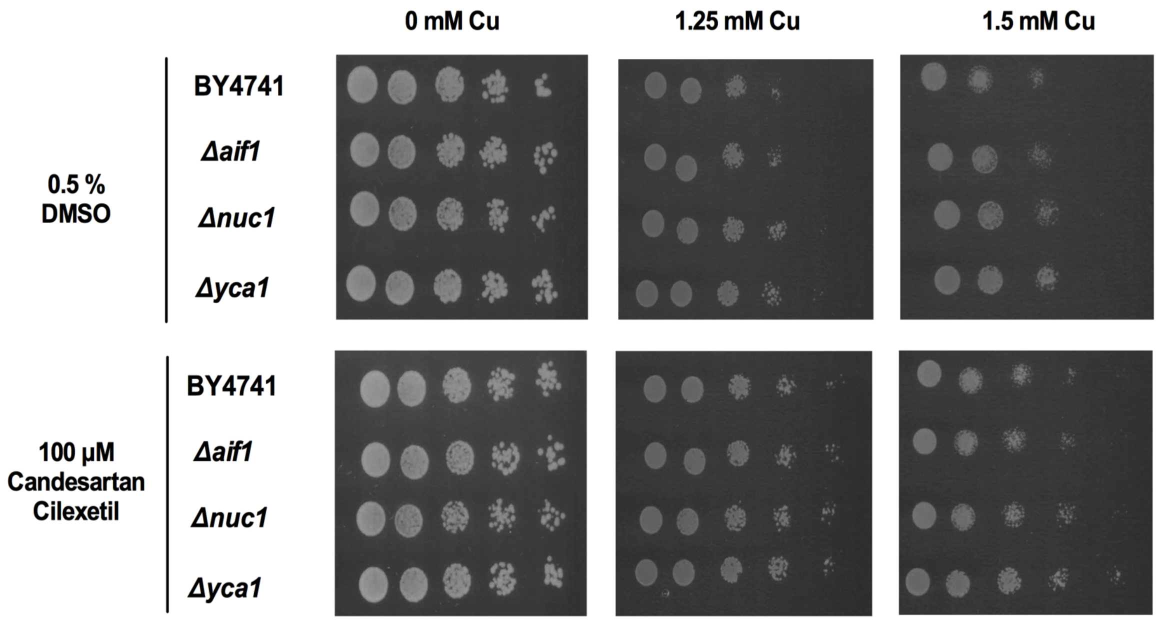

Supplement: Supplementary file 1 [file mic-01-352-s01.pdf]
